# CGRP-mediated neuro-vascular-pulp cell crosstalk is essential for dental pulp repair

**DOI:** 10.3389/fcell.2026.1793692

**Published:** 2026-04-21

**Authors:** Zixin Wu, Junyang Chen, Yiming Zhao, Zhenjuan Zhou, Rui Lin, Wei Liu

**Affiliations:** 1 Shenzhen Stomatology Hospital (Pingshan) of Southern Medical University, Shenzhen, China; 2 Stomatology Center, Shenzhen Hospital, Southern Medical University, Shenzhen, China; 3 Second Department of Oral and Maxillofacial Surgery, Graduate School of Dentistry, Osaka Dental University, Hirakata, Osaka, Japan; 4 Department of Stomatology, Shenzhen Hospital, Southern Medical University, Shenzhen, China; 5 Shenzhen Clinical College of Stomatology, School of Stomatology, Southern Medical University, Shenzhen, China

**Keywords:** calcitonin gene-related peptide, dental pulp cells, endothelial cells, neuropeptide, pulp biology

## Abstract

**Introduction:**

The repair of dental pulp injury is the cornerstone of vital pulp therapy. Traditional research has predominantly focused on the roles of immune cells, vascular endothelial cells, and dental pulp stem cells, often overlooking the active regulatory functions of the sensory neural network. Sensory nerve fibers constitute nearly 40% of the dental pulp volume, and their released neuropeptides, such as calcitonin Gene-Related Peptide (CGRP), are hypothesized to coordinate the repair process via intercellular communication. This study aims to systematically elucidate the molecular mechanisms by which sensory nerves and their key neuropeptide, CGRP, regulate angiogenesis and the activation of stromal cells within the injured pulp microenvironment, thereby providing a theoretical basis for novel pulp regeneration strategies targeting neural signaling pathways.

**Methods:**

The cell-cell communication network in dental pulp was using single-cell transcriptome analysis (GSE197289, GSE274562, GSE280528). A series of in vitrocellular experiments, including qPCR, Western blot, immunofluorescence, scratch wound healing assay, and tube formation assay, were employed to evaluate the effects of CGRP on cell migration, angiogenesis, and mineralization. A mouse model of dentin-pulp injury was established, and the in vivoangiogenic changes were validated through intervention with the CGRP receptor antagonist BIBN4096BS. RNA sequencing was conducted to analyze the transcriptional reprogramming of human dental pulp cells (DPCs) induced by CGRP.

**Results:**

Single-cell communication analysis revealed intensive CGRP signaling interactions between sensory neurons, endothelial cells, and dental pulp cells. In vitroexperiments demonstrated that CGRP directly enhanced the tube-forming and migratory capabilities of human umbilical vein endothelial cells (HUVECs) and upregulated the expression of CD31/VEGFA. Furthermore, CGRP potentiated the mineralization of dental Pulp Stem Cells (DPSCs) via a paracrine mechanism. Concurrently, CGRP significantly accelerated the migration of DPCs, and the conditioned medium from CGRP-pretreated DPCs enhanced endothelial tube formation, a mechanism involving the upregulation of VEGFA and the activation of IL-17/TNF signaling pathways. In vivoexperiments confirmed that inhibition of CGRP signaling significantly reduced angiogenesis (CD31^+^ signals) in the injured pulp area of mice.

**Conclusion:**

This study elucidates that the sensory neuropeptide CGRP drives a “neuro-vascular-stromal cell” collaborative network during pulp repair through dual pathways: directly activating endothelial cell function and indirectly modulating the paracrine profile of dental pulp cells (e.g., upregulating VEGFA), thereby promoting angiogenesis and stem cell differentiation. This discovery not only deepens the understanding of the self-repair mechanisms of dental pulp but also offers a new perspective for developing precise vital pulp therapy strategies targeting CGRP signaling, such as modulating the neuro-microenvironment interaction.

## Introduction

The dental pulp, as the core component of tooth structure, not only provides nutritional, sensory, and defensive functions but also exhibits remarkable self-repair potential (Mitsiadis and Rahiotis, 2004). Vital pulp preservation represents a fundamental concept in modern endodontic therapy, the success of which largely depends on a deep understanding of the innate repair mechanisms of the pulp (Fujino et al., 2023). Traditionally, research on pulp injury repair has primarily focused on angiogenesis, immune-inflammatory responses, and stem cell differentiation, while overlooking a critical component: the sensory neural network (Grutzner et al., 1992). The dental pulp contains an abundant innervation, with sensory nerve fibers accounting for nearly 40% of its volume (França et al., 2019). These nerve fibers originate mainly from the trigeminal ganglion, enter the pulp chamber through the apical foramen, accompany blood vessels to form complex neurovascular bundles, and branch into a dense network (Matsubara et al., 2022). In the central pulp, nerve fibers are relatively large, whereas in the coronal region, they branch into finer fibers terminating in the odontoblastic layer or dentinal tubules (Wak and isaka, 1990). This unique anatomical distribution enables dental pulp nerves to rapidly perceive external stimuli, serving as the first line of defense (Kim, 1990). Notably, dental pulp nerves are not merely passive signal conductors. Growing evidence indicates that neural tissues play active regulatory roles in the injury repair of various tissues, such as bone, skin, and cornea (Crane et al., 2024; Lu et al., 2024). In fracture models, sensory nerves sprout rapidly towards the injury site (Mi et al., 2021), and their deficiency impairs bone healing (Xu et al., 2020). Similarly, the absence of sensory nerves significantly delays healing in skin and corneal injury models (Lu et al., 2024; Okada et al., 2025). These findings suggest that sensory nerves in the dental pulp likely act as an “invisible conductor” in pulp repair.

Sensory nerves primarily exert their regulatory functions by releasing various neuropeptides, among which calcitonin Gene-Related Peptide (CGRP) is one of the most extensively studied (Russell et al., 2014). CGRP, a major neuropeptide released from sensory nerve terminals, shows significantly increased expression levels in response to chemical, thermal, or mechanical stimulation of the pulp. Research indicates that CGRP possesses multiple biological functions; it is one of the most potent vasodilators known, and approximately 20% of dental pulp blood vessels demonstrate positive innervation. Among those nerves associated with blood vessels, CGRP-positive nerve fibers are densely distributed and show a close anatomical association with blood vessels in the dental pulp (Caviedes-Bucheli et al., 2021). During pulp injury, CGRP is involved not only in vasodilation and inflammation regulation but may also directly influence the biological behavior of pulp tissues. Studies have identified sensory nerves as key upstream mediators promoting angiogenesis (including H-type vessel formation) and reparative dentinogenesis, suggesting CGRP signaling controls a neuro-vascular-reparative dentin network (Zhan et al., 2024). However, some reports indicate that CGRP might inhibit the proliferation and mineralization of dental Pulp Stem Cells (DPSCs), suggesting its effects could be context-dependent and concentration-specific (Michot et al., 2020). Recent advances in single-cell transcriptomics provide new insights into the mechanism of CGRP. Studies show that CGRP and its receptor component RAMP1 are among the highest expressed ligand-receptor pairs between sensory nerves and DPSCs (Wang et al., 2024). By activating downstream signaling pathways (e.g., CREB phosphorylation), CGRP can regulate stem cell migration, thereby influencing the pulp repair process. Though existing research suggests sensory nerves and CGRP play important roles in pulp repair, significant knowledge gaps remain regarding how sensory nerves regulate the pulp microenvironment via CGRP-mediated intercellular communication networks and the specific mechanisms of this network in injury repair (Moore et al., 2022).

Although sensory nerve-derived CGRP is known to promote dental pulp stem cells (DPSCs) migration, its effects on the major stromal and vascular components of the pulp are poorly understood. This study investigates the hypothesis that CGRP critically coordinates a ‘neuro-stromal-vascular’ axis​ during pulp repair. By integrating single-cell transcriptomic analysis of human pulpitis, *in vitro* functional assays on dental pulp cells (DPCs) and endothelial cells, and animal model validation, we systematically define the mechanisms by which CGRP regulates key cell types beyond DPSCs. We focus on elucidating how CGRP modulates DPCs and endothelial cells to drive vascularization and matrix remodeling. This work aims to uncover new dimensions of ‘neuro-vascular-odontogenic’ crosstalk, offering novel insights for neural modulation-based therapeutic strategies in vital pulp therapy.

## Materials and methods

### Materials

HUVECs were purchased from Saiye Co. (China). CGRP (human) was purchased from KKL Med Co. (cat. #90954-53-3). Bicinchoninic acid (BCA) assay kit were obtained from Beyotime Biotechnology Co. (Jiangsu, China). Alizarin red sodium salt was obtained from Alfa Aesar Co. (Tianjin, China). The CGRP receptor antagonist BIBN4096BS was purchased from Shanghai Haoyuan Chemexpress Co. (Shanghai, China). Phosphate buffer solution (PBS) was provided by Dingguo Biotechnology Co. (Beijing, China). Other chemicals were purchased from Oriental Chemical Co. (Chongqing, China).

### Animals and surgeries

All animal procedures were approved by the Ethics Committee of Southern Medical University (IACUC-LAC-20230607-003) and conducted in compliance with ARRIVE 2.0 guidelines. A dentine injury model was established in the first molars of male C57BL/6J mice (8–9 weeks old) as per the previously described protocol (Zhan et al., 2024). Briefly, anesthetized mice with ketamine (100 mg/kg) and xylazine (10 mg/kg), underwent preparation of a groove shaped cavity on the mesial aspect of first molars using a 1/4 round carbide bur. To create a standardized dentin exposure (Zhan et al., 2024), the exposed dentine was etched with 37% phosphoric acid for 3 min. This model induces a localized dentin injury without direct pulp exposure, aimed at studying the reparative response of the pulp-dentin complex. CGRP pathway intervention: 24 mice were assigned to 7-day subdivided into NC or CGRP receptor antagonist, Olcegepant (BIBN4096, HY-10095). BIBN4096 was administered intravenously (0.1 mg/kg) pre injury and intraperitoneally (1 mg/kg/day) for 7 days post-injury. Mice were transcardially perfused with 0.9% saline followed by 4% paraformaldehyde. Tissues were decalcified in 0.5 M EDTA for 3 weeks. For immunofluorescence (IF), tissue sections were cryosectioned at a thickness of 20–40 μm and the cryosections were permeabilized (0.5% Triton X-100, 5 min) and blocked (10% goat serum, 1 h, RT). Sections were incubated overnight at 4 °C with primary antibodies (anti-CD31/Platelet endothelial cell adhesion molecule-1, Abcam, ab182981; rabbit monoclonal; dilution 1:100), and incubated (1 h, RT) with 1:1,000 secondary antibodies (Alexa Fluor 647 goat anti-rabbit; Abcam, ab150083), followed by DAPI counterstaining. IF images were acquired via fluorescence microscopy (Nikon Eclipse Ts2-FL) or confocal microscopy (ZEISS LSM980). Confocal images consisted of merged 40-section Z-stacks. Vessels were quantified from maximum intensity projections using the ImageJ VesselJ plugin in a blinded manner.

### Cell culture

Commercially available human dental pulp stem cells (DPSCs) (Lonza) were cultured in either DPSC Growth Medium (Lonza) or α-Minimum Essential Medium (α-MEM; Sigma-Aldrich) supplemented with 10% fetal bovine serum (FBS), 2 mmol/L L-glutamine, 100 mmol/L ascorbic acid, 100 U/mL penicillin, 100 μg/mL streptomycin, and 2.5 μg/mL amphotericin B. Human dental pulp cells (DPCs) were isolated from extracted healthy human premolars and third molars. Briefly, the pulp tissue was digested with 3 mg/mL type I collagenase (Gibco) at 37 °C for 1 h. The resulting cell suspension was seeded into culture flasks and maintained in DMEM (Corning) supplemented with 10% FBS (Corning), 100 U/mL penicillin, and 100 μg/mL streptomycin at 37 °C in a 5% CO_2_ atmosphere. The culture medium was changed every 2 days. Informed consent was obtained from all patients, and the study protocol was approved by the Ethics Committee. Both DPCs (at passage 3) and human umbilical vein endothelial cells (HUVECs) were routinely cultured in tissue culture polystyrene (TCPS) dishes in DMEM supplemented with 10% FBS, 100 U/mL penicillin, and 100 μg/mL streptomycin. Cells were passaged using 0.05% trypsin. Cells were passaged upon reaching 85% confluence, and cells between passages 3 and 5 were used for experiments.

### Immunostaining

DPCs were treated for 24 h with or without CGRP (10^−8^ mol/L). Cells were fixed with 10% formalin for 15 min, washed in PBS, and incubated in blocking solution (PBS 1 1% triton 1 1% bovine serum albumin) for 1 h at room temperature. Cells were incubated overnight at 4 C with anti–VEGFA primary antibody (rabbit monoclonal; dilution 1:100, Invitrogen, Carlsbad, CA, 7422-RBM5-P1ABX). Cells were washed with a blocking solution, incubated for 1 h at room temperature with Alexa Fluor 647–conjugated secondary antibody (Goat anti-Rabbit, 1:1000; Invitrogen, Carlsbad, CA, A-21245), washed with PBS, and visualized using a fluorescent microscope.

### Quantitative real-time polymerase chain reaction (qRT-PCR) assay

Gene expression levels of target genes in human dental pulp cells (DPCs) and human umbilical vein endothelial cells (HUVECs) were analyzed by quantitative real-time PCR (qRT-PCR). Total RNA was extracted using Trizol reagent, and first-strand cDNA was synthesized using a commercial RNA extract kit and the PrimeScript™ RT reagent kit. qPCR amplification was performed on a Bio-Rad CFX Manager system under the following conditions: initial denaturation at 95 °C for 30 s, followed by 39 cycles of 95 °C for 5 s and 60 °C for 30 s. The primer sequences used for amplification were as follows: CD31 forward, 5′-GAG​TCC​AGC​CGC​ATA​TCC​AA-3′ and reverse, 5′-GGA​GCA​GGA​CAG​GTT​CAG​TC-3'; VEGFA forward, 5′-CCT​TGC​CTT​GCT​GCT​CTA​CCT​C-3′ and reverse, 5′-GAT​GAT​TCT​GCC​CTC​CTC​CTT​CTG-3'; TGFB forward, 5′-CGC​CAG​AGT​GGT​TAT​CTT​TTG-3′ and reverse, 5′-CGG​TAG​TGA​ACC​CGT​TGA​TGT-3'; BMP2 forward, 5′-ACT​CGA​AAT​TCC​CCG​TGA​CC-3′ and reverse, 5′-CCA​CTT​CCA​CCA​CCA​CGA​ATC​CA-3'. GAPDH (forward, 5′-CCC​ACC​ACA​CTG​AAT​CTC​CC-3′ and reverse, 5′-TGG​TAC​ATG​ACA​AGG​TGC​GG-3′) was used as the reference gene for normalization.

### Western blotting

For Western blotting experiments, the HUVECs were lysed with Laemmli sample buffer (Bio-Rad Laboratories, CA, United States). The total protein content of the lysates was measured using the Pierce BCA Protein Assay Kit (ThermoFisher Scientific-Life Technologies, Carlsbad, CA, United States), followed by further heating at 100 °C for 10 min. The samples were loaded onto 12% sodium dodecyl sulfate-polyacrylamide gels (SDS-PAGE) with equal amounts of total protein, electrotransferred to polyvinylidene fluoride (PVDF) membranes (Bio-Rad Laboratories, Inc.), and blocked with 1X TBST with 5% *w*/*v* nonfat dry milk at room temperature for 1.5 h. The membranes were incubated with primary antibodies overnight at 4 °C. Rabbit monoclonal anti-CD31 (Platelet endothelial cell adhesion molecule-1, 1:1000, Proteintech, 11265-1-AP), anti-VEGFA (Vascular endothelial growth factor A, 1:1000, Abacm, ab214424), and anti-GADPH (glyceraldehyde 3-phosphate dehydrogenase, 1:1000, Abacm, ab8245) were used. The blots were then exposed to the horseradish peroxidase-labeled secondary antirabbit antibody (Goat Anti-Rabbit IgG H&L HRP, 1:5000; Abcam, ab6721) for 2 h at room temperature. The proteins were visualized using an enhanced chemiluminescence kit (Thermo Fisher Scientific, MA, United States), and the band signals were detected using a gel imaging system (Syngene, Frederick, MD, United States). The intensities were quantified using ImageJ software, and the relative expression levels of certain proteins were calculated via band intensity normalization.

### Tube formation assays

HUVECs (5.0 × 10^4^) were seeded on growth factor-reduced Matrigel Matrix (Corning, United States) coated wells in a 96-well plate with or without CGRP for 24 h. Tube formation was observed with an inverted microscope, and images of random separate fields from each group were recorded. To investigate the paracrine effects of DPCs on tube formation of HUVECs, DPCs-derived conditioned medium (CM) was firstly obtained from DPCs cultures. A total of 1.6 × 10^5^ DPCs per well were firstly seeded onto 24-well plates and incubated for 3 days with or without CGRP. After being washed 3 times with phosphate-buffered saline (PBS), the medium was replaced with serum-free DMEM for 24 h. The CM was concentrated and used as the HUVECs culture medium. HUVECs tube formation assay was performed as mentioned above.

### Wound healing assay

To investigate the *in vitro* migration of HUVECs and DPCs, a scratch wound assay model was applied. In short, they were seeded in 24-well plates at a density of 1.0 × 10^5^/well. After reaching 90% confluency, the cell layers were then scratched longitudinally with a pipette tip to create a straight wound. The samples were washed with PBS for two times, and the remained cells were further cultured in medium supplemented with 1% FBS for 12 h. The wound area of HUVECs was observed with an inverted microscope at 0 and 12/24 h, and images of random separate fields from each group were recorded. The wound area was calculated by manually tracing the cell-free area in captured images using Image-Pro Plus software (Media Cybernetics). The relative wound closure was expressed as the percentage of wound area change over time.

### RNA sequencing (RNA-seq) analysis

DPCs was stimulated with 10^−8^ mol/L CGRP for 24 h, and total RNA was harvested using Trizol reagent (Invitrogen, CA, United States). RNA purity and quantification were assessed using a NanoDrop 2000 spectrophotometer (Thermo Scientific, United States). RNA integrity was assessed using the Agilent 2100 bioanalyzer (Agilent Technologies, Santa Clara, CA, United States). The library was then constructed using the VAHTS universal V6 RNA-seq Library preparation kit according to the manufacturer’s instructions. Transcriptome sequencing and analysis were performed by OE Biotech Co., Ltd. (Shanghai, China). Foldchange <0.5 were set as thresholds for significantly differently-expressed genes (DEGs). Based on hypergeometric distribution, KEGG 7 pathway enrichment analysis of DEGs was performed using R (v3.2.0), and significant enrichment items were screened.

### Re-analysis of scRNA-seq data

To investigate the potential crosstalk between sensory neurons and dental pulp cells, as well as the changes during inflammation, we integrated scRNA-seq data from both neuronal and pulp tissues sourced from the Gene Expression Omnibus (GEO) database. For neuron-pulp interaction analysis: Data from the human trigeminal ganglion (GSE197289) and healthy human dental pulp (GSE274562) were integrated. The single-cell RNA sequencing (scRNA-seq) data for integration of human trigeminal ganglion (GSE197289) and healthy human dental pulp (GSE274562) utilized in this study were sourced from the Gene Expression Omnibus (GEO) database. Raw sequencing data were processed using Cell Ranger (version 3.1.0) to generate gene expression matrices. Subsequent downstream analyses were performed in R using the Seurat package (version 5.0.0). Briefly, cells were filtered, and gene expression counts were normalized. Dimensionality reduction was conducted via principal component analysis (PCA), followed by graph-based clustering. Cell clusters were visualized in two dimensions using Uniform Manifold Approximation and Projection (UMAP). Each major cell type cluster was annotated based on the average expression of well-established marker gene sets. To explore potential ligand-receptor interactions between cell types, cell-cell communication analysis was performed using CellPhoneDB (version 5.0.0). For inflammatory pulp analysis: To compare cellular landscapes between states, we analyzed data from healthy control (GSE274562) and pulpitis (GSE280528) samples. The scRNA-seq data for comparative analysis between healthy and inflamed dental pulp were obtained from datasets (healthy control: GSE274562; pulpitis: GSE280528). The data processing and analysis pipeline, including matrix generation with Cell Ranger, quality control, normalization, clustering, visualization with UMAP, and cell type annotation, was identical to that described in the preceding section for trigeminal ganglion and dental pulp integration. Cell-cell communication analysis between conditions was likewise performed using CellPhoneDB.

### Paracrine effects of HUVECs on mineralization assay

To investigate the paracrine effects of HUVECs on mineralization of DPSCs, HUVECs-derived conditioned medium (CM) was firstly obtained from HUVECs cultures. A total of 1.6 × 10^5^ HUVECs per well were firstly seeded onto 24-well plates and incubated for 3 days with or without CGRP. After being washed 3 times with phosphate-buffered saline (PBS), the medium was replaced with serum-free DMEM for 24 h. The CM was concentrated and used as the DPSCs culture medium. The DPSCs (2 × 10^4^ cells/cm^2^) were firstly seeded onto 24-well plates. The Control group was incubated with DMEM based culture medium only as mentioned above. The experimental groups were treated DMEM based culture medium plus 10^−8^ mol/L CGRP for the CGRP group, and its HUVECs-derived conditioned medium for the CM group. The ALP staining and activity assay of DPSCs were performed after culture for 7 days. For ALP staining, DPSCs were fixed with 4% paraformaldehyde for 30 min and stained with the BCIP/NBT alkaline phosphatase staining kit, respectively. Then, images were recorded using an Olympus MVX10 MacroView (Japan). The mineralization assay was performed according to a previous study. Specifically, after 14 days of culture, the mineralization of DPSCs (initial density of 2 × 10^4^ cells/mL) was investigated via Alizarin Red S staining (ARS). Alizarin Red S is considered as the gold standard for assessing calcium deposits in the mineralized extracellular matrix (ECM) of differentiated stem cells. ARS works through a chelation process during which calcium forms an Alizarin Red S-calcium complex, and the stain is directly proportional to the degree of mineralization. Briefly, DPSCs, rinsed with PBS 3 times, were fixed with 4% paraformaldehyde and stained with 40 mM Alizarin Red S (pH4.1), respectively. The staining images were recorded using an Olympus MVX10 MacroView (Japan).

### Data analysis

All data were present as means ± standarddeviation (SD). Statistical analyses were performed using GraphPad Prism software (version 5.0, GraphPad Software Inc., San Diego, CA). For comparisons between two groups, an unpaired two-tailed Student’s t-test was used. For comparisons among three or more groups, one-way analysis of variance (ANOVA) was performed, followed by Dunnett’s *post hoc* test for comparisons against a single control group. The confidence levels were set as 95% and 99%.

## Results

### The role of sensory nerves in pulp injury healing: a study on CGRP-mediated intercellular communication

**FIGURE 1:**
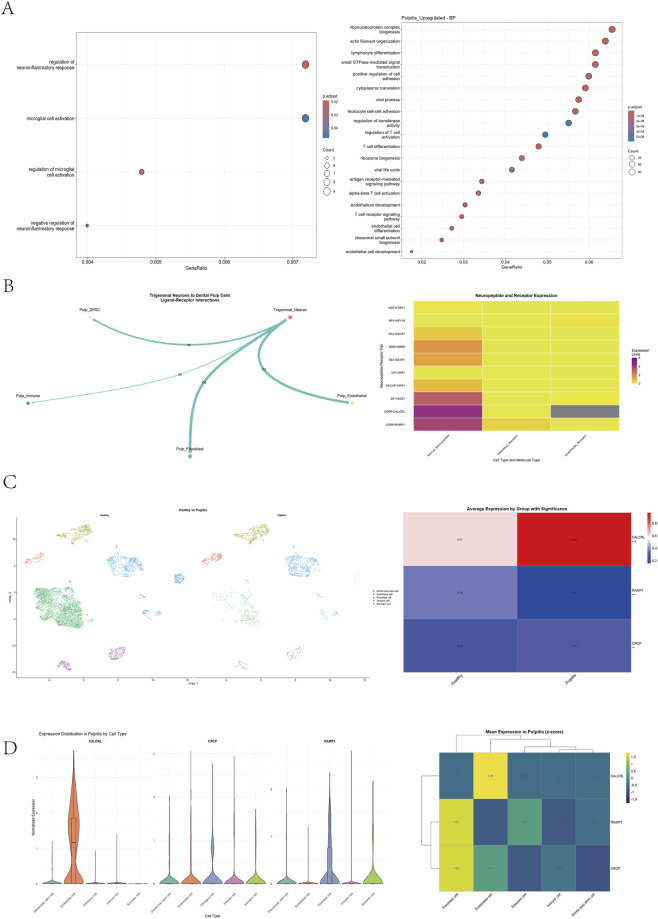
Sensory nerve-derived CGRP signaling is activated and implicated in intercellular communication during pulp injury and inflammation. **(A)** Pathway enrichment analysis of differentially expressed genes from RNA sequencing of pulpitis versus normal pulp tissues. **(B)**​ Re-analysis of public single-cell RNA sequencing data (GSE197289, GSE274562). *Left:*Cell-cell communication network inferred by Cellchat, highlighting interactions between neurons, endothelial cells, and dental pulp cells. *Right:*Heatmap displaying the expression levels of key neuropeptides, including CGRP, in sensory neurons and the receptor–ligand pairs identified by CellPhoneDB between neurons and other cell types. **(C)**​ UMAP plot of cell clusters from human pulpitis tissue and plot showing upregulated expression of the CGRP receptor component in pulpitis compared to healthy pulp. **(D)**​ Plots showing the levels of upregulated expression of the CGRP receptor component in pulpitis in various cell types.

This study aimed to investigate the mechanism of action of sensory nerves, particularly their released neuropeptides, in the process of dental pulp injury and inflammation. Both human and mouse dental pulp nerves are predominantly composed of sensory nerve fibers. These fibers bundle within the root canal and branch into a dense network in the coronal pulp chamber, significantly enhancing the pulp’s sensitivity to external stimuli. To analyze the molecular response after pulp injury, RNA sequencing was performed on normal (GSE274562) and injured pulpitis tissues (GSE280528). Enrichment pathway analysis revealed that post-injury genes were significantly enriched in pathways related to the regulation of neuroinflammation and vascular development (Figure 1A), suggesting a potential synergistic role between the nervous and vascular systems in injury repair. Based on previous reports indicating significant changes in genes related to sensory nerve function after pulp injury, we hypothesized that sensory nerves might actively participate in the pulp healing process. To test this hypothesis, we integrated and re-analyzed single-cell RNA sequencing data from the human trigeminal ganglion (GSE197289) and human healthy dental pulp (GSE274562). Using canonical marker genes, we successfully identified peripheral glial cells (Schwann cells), endothelial cells, dental pulp cells (fibroblasts), and other cell types (Figure 1B). Further construction of cell-cell interaction networks using the Cellchat tool revealed close communication between neurons and endothelial cells, as well as between neurons and dental pulp cells (Figure 1B). Notably, multiple neuropeptide-related ligand-receptor interactions were identified specifically between neuron-endothelial cell and neuron-pulp cell pairs (Figure 1B). Heatmap analysis showed significantly high expression of the sensory neuropeptide CGRP in the trigeminal ganglion (Figure 1B), suggesting that sensory nerves originating from the trigeminal ganglion might regulate the biological functions of endothelial cells and pulp cells within the pulp via CGRP release. This finding aligns with previous research by Hou et al., indicating that sensory nerves promote angiogenesis (including H-type vessel formation) in dentin-pulp injury through the CGRP signaling pathway, thus constituting a “neuro-vascular” regulatory network. To further explore the role of CGRP in pulp inflammation, we performed single-cell transcriptome analysis on human pulpitis tissue (GSE280528). We successfully identified major cell subsets, including peripheral glial cells (Schwann cells), dental pulp stem cells, endothelial cells, immune cells and fibroblast cells (Figure 1C). Analysis of cellular composition revealed a significant shift in cell type proportions during pulpitis. Notably, the proportion of immune cells increased substantially, while the proportion of fibroblast cells markedly decreased. The proportions of dental pulp stem cells and endothelial cells also increased. To investigate the potential for CGRP signaling within the pulpitis microenvironment, we analyzed the single-cell expression profiles of the core molecular components of the canonical CGRP receptor complex. This included the Calcitonin Receptor-Like Receptor (CALCRL), the Receptor Activity-Modifying Protein 1 (RAMP1) which is essential for forming the high-affinity CGRP receptor, and the Receptor Component Protein (CRCP) which is crucial for efficient downstream signal transduction of this receptor. Comparative analysis revealed a selective upregulation of the Calcitonin Receptor-Like Receptor (CALCRL) subunit in pulpitis tissue, while the expression levels of its essential co-receptor RAMP1 and the signal transduction component CRCP did not show a significant increase (Figure 1C). These results indicate a specific alteration in the expression profile of CGRP receptor components​ during pulp inflammation. Subsequent cellular analysis indicated that CGRP signaling primarily participates in the downstream biological processes of pulpitis by regulating endothelial cells and dental pulp cells (Figure 1D). In summary, our integrated sequencing analysis unveils the key regulatory role of sensory nerves and their neuropeptide CGRP in pulp injury repair and inflammatory responses, potentially by modulating endothelial cells and pulp cells involved in pulpitis.

### CGRP promotes angiogenesis in HUVECs and pulp repair via upregulation of CD31/VEGFA

**FIGURE 2:**
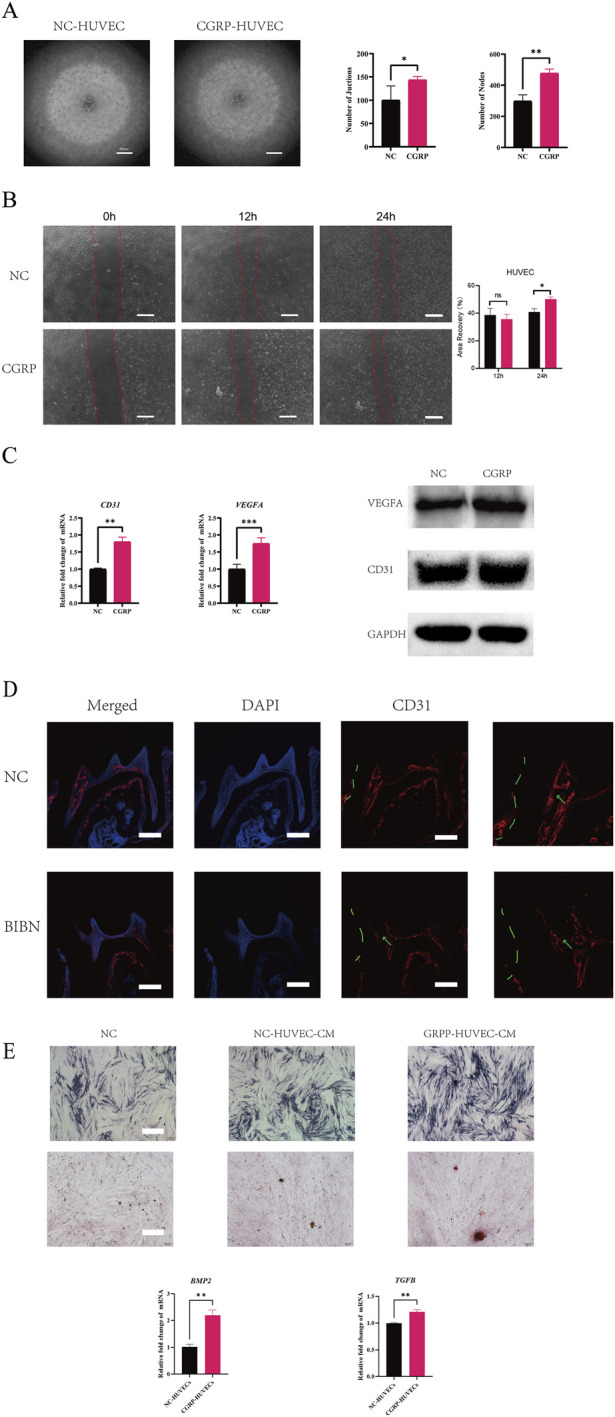
CGRP Promotes Angiogenesis in HUVECs and Pulp Repair via Upregulation of CD31/VEGFA **(A)** Representative images of tube formation assays using HUVECs treated with or without CGRP. Scale bar, 500 µm. (n = 3, *p < 0.05, **p < 0.01 vs. NC group). **(B)**​ Representative images of scratch wound healing assays of HUVECs at 0,12 and 24 h post-CGRP treatment and quantitative analysis of wound closure rate. Scale bar, 100 µm. (n = 3, *p < 0.05 vs. NC group). **(C)**​ qRT-PCR and Western blot analysis of CD31 and VEGFA levels in HUVECs. (n = 3, **p < 0.01, ***p < 0.001 vs. NC group). **(D)**​ Representative immunofluorescence images showing CD31^+^ blood vessels (red) in the injured pulp region of mice treated with either vehicle (NC) or the CGRP receptor antagonist BIBN4096BS. Nuclei are counterstained with DAPI (blue). Scale bar, 50 µm. Green dashed line, dentin injury (cavity). Green arrow, region of interest (ROI) in the pulp horn beneath the injury site, where vascular changes were analyzed. **(E)**​ CGRP-activated endothelial cells promote the mineralization of DPSCs via a paracrine mechanism. (n = 3, **p < 0.01vs. NC group).

To elucidate the pro-angiogenic role of CGRP in pulp repair, we first conducted a series of functional experiments at the cellular level. As shown in Figure 2A, CGRP-treated human umbilical vein endothelial cells (HUVECs) formed richer and more mature tubular network structures *in vitro*, indicating a significant pro-angiogenic capability. Furthermore, a scratch wound assay demonstrated that CGRP markedly accelerated the migration of HUVECs, significantly promoting wound closure within 24 h (Figure 2B), suggesting CGRP effectively activates the motility and repair functions of endothelial cells. To decipher the underlying molecular mechanism, we examined the expression changes of key angiogenesis-related molecules. qRT-PCR and Western blot results showed that CGRP treatment significantly upregulated the expression of both the canonical endothelial marker CD31 (PECAM-1) and the potent angiogenic growth factor VEGFA at transcriptional and protein levels (Figure 2C). This coordinated induction suggests that CGRP promotes angiogenesis by concurrently enhancing endothelial cell identity/activity (reflected by CD31) and amplifying the key signal of vasculogenesis (VEGFA). To validate the relevance of these *in vitro* findings in a physiological context, we performed *in vivo* analysis using a mouse model of dentin-pulp injury. Immunofluorescence staining revealed a significant increase in CD31 expression in the local pulp tissue at the injury site, suggesting active angiogenesis accompanies injury repair. This increased CD31 signal was significantly attenuated upon treatment with the CGRP receptor antagonist BIBN4096BS (Figure 2D), further confirming the critical role of CGRP signaling in the vascularization process following pulp injury. Interestingly, although CGRP did not directly promote the odontogenic differentiation of dental Pulp Stem Cells (DPSCs), we found it could indirectly influence the repair process by modulating endothelial cells. Conditioned medium from CGRP-pretreated HUVECs (CGRP-HUVEC-CM) significantly enhanced the mineralization capacity of DPSCs (Figure 2E). Consistent with this, qRT-PCR analysis indicated that CGRP stimulation induced the upregulation of pro-mineralization factors BMP2 and TGFβ in HUVECs (Figure 2E). These results suggest that CGRP-activated endothelial cells can release pro-mineralization factors in a paracrine manner, thereby regulating the odontogenic/osteogenic differentiation of DPSCs, thus synergistically promoting angiogenesis and hard tissue formation in pulp injury repair. In summary, our study systematically demonstrates from cellular, molecular, and animal model perspectives that CGRP not only directly promotes the angiogenic activity of endothelial cells but also indirectly enhances the mineralization capacity of DPSCs by modulating the secretory profile of endothelial cells, potentially playing a key role in the “neuro-vascular-odontogenic” crosstalk during pulp injury repair.

### CGRP promotes dental pulp cell migration, upregulates VEGFA expression, and enhances endothelial tubulogenesis via paracrine action

**FIGURE 3:**
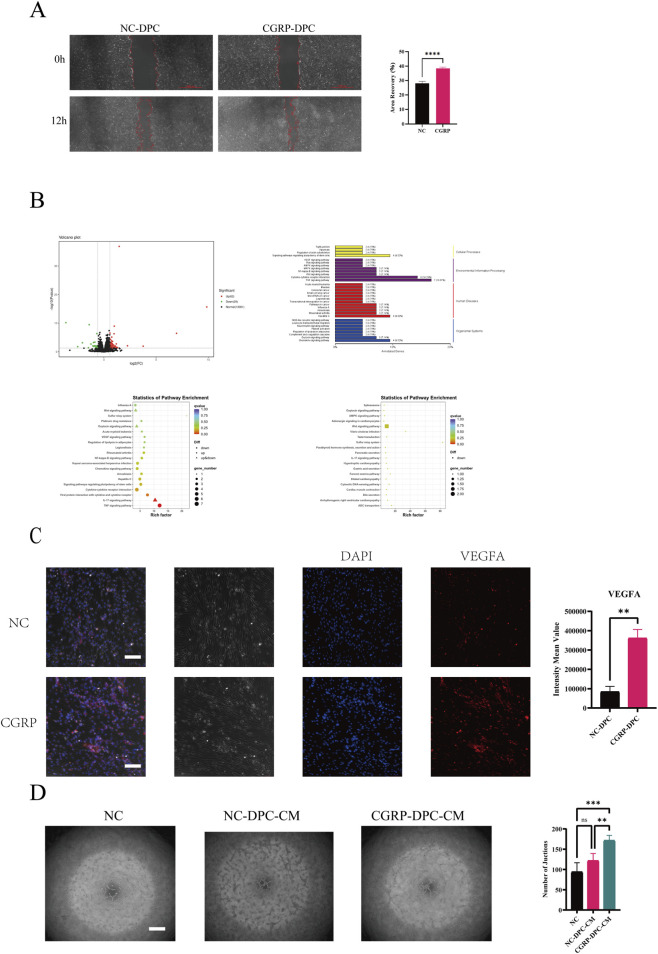
CGRP enhances the migration and pro-angiogenic paracrine function of dental pulp cells (DPCs). **(A)** CGRP promotes DPCs migration. *Left:*Representative images of scratch wound healing assays of DPCs at 0 and 12 h. Scale bar, 100 µm. *Right:*Quantitative analysis of wound closure rate (n = 3, ****p < 0.0001 vs. NC group). **(B)**​ Transcriptomic profiling of CGRP-treated DPCs. Volcano plot displaying differentially expressed genes (DEGs) in CGRP-treated vs. control DPCs and Gene Ontology (GO) and Kyoto Encyclopedia of Genes and Genomes (KEGG) pathway enrichment analysis of the DEGs. **(C)**​ Representative immunofluorescence images showing VEGFA (red) expression in DPCs with or without CGRP treatment. Nuclei are stained with DAPI (blue). Scale bar, 20 µm.(n = 3, **p < 0.01vs. NC group). **(D)**​ Conditioned medium from CGRP-pretreated DPCs enhances HUVEC tubulogenesis. Representative images of tube formation assays using HUVECs cultured with control medium (NC), conditioned medium from untreated DPCs (NC-DPC-CM), or conditioned medium from CGRP-treated DPCs (CGRP-DPC-CM). Scale bar, 500 µm. (n = 3, **p < 0.01, ***p < 0.001 vs. NC group)

This section focuses on the regulatory effect of the sensory neuropeptide CGRP on the function of dental pulp cells/fibroblasts (DPCs), the core stromal cells of the pulp. As shown in Figure 3A, a scratch wound healing assay confirmed that CGRP treatment significantly enhanced the migration ability of DPCs, with wound closure occurring faster in the CGRP-treated group compared to the control group (NC-DPC group) at the 12-h time point, indicating CGRP is an effective regulator of DPC motility. To systematically analyze the transcriptomic basis of the phenotypic changes induced by CGRP in DPCs, we performed RNA sequencing on CGRP-treated DPCs. The volcano plot revealed a specific transcriptional reprogramming triggered by CGRP stimulation compared to the vast majority of unchanged genes, identifying 62 significantly upregulated and 29 significantly downregulated genes (Figure 3B). This limited yet distinct differentially expressed gene profile suggests that CGRP induces a targeted rather than generalized transcriptional response in DPCs. Gene Ontology (GO) enrichment analysis indicated that these differentially expressed genes were significantly enriched in functional terms related to cell migration, movement, and cytoskeletal reorganization (Figure 3B). Further KEGG pathway analysis provided mechanistic insights for this phenotype: the most significantly enriched pathways included the IL-17 signaling pathway, TNF signaling pathway, chemokine signaling pathway, and cytokine-cytokine receptor interaction (Figure 3B), all known to regulate cytoskeletal dynamics and cell migration via downstream effectors like MAPK and NF-κB. Notably, the VEGF signaling pathway was also significantly activated (Figure 3B), prompting us to investigate whether CGRP affects angiogenesis by modulating the secretory profile of DPCs. Based on these clues, we first examined changes in key pro-angiogenic factors. Immunofluorescence staining showed that CGRP treatment significantly upregulated the expression level of VEGFA in DPCs (Figure 3C). To validate the functional consequence of this molecular change, we further investigated the effect of DPC-conditioned medium on the tubulogenesis ability of endothelial cells. A tube formation assay demonstrated that conditioned medium from CGRP-stimulated DPCs significantly promoted the formation of richer and more mature tubular networks by HUVECs (Figure 3D), whereas the effect of the control conditioned medium was weaker. This result directly confirms that CGRP can enhance the paracrine pro-angiogenic capacity of DPCs. In conclusion, this part of the study elucidates that CGRP not only directly promotes the migration of DPCs but also, by reprogramming their transcriptional profile, particularly upregulating factors like VEGFA, transforms DPCs into an “activated” stromal cell type that enhances endothelial angiogenesis via a paracrine mechanism. This provides new evidence for understanding the “cell migration-angiogenesis” cooperative network regulated by sensory nerves in pulp repair.

## Discussion

The dental pulp is a unique loose connective tissue encased by hard dentin. Its repair process following injury is a highly complex and orderly physiological event involving precise regulation at cellular, molecular, and microenvironmental levels (Sismanoglu et al., 2020). Traditionally, research has primarily focused on the central roles of immune cells, vascular endothelial cells, and dental pulp stem cells (DPSCs) in this process (Hashemi-Beni et al., 2017). Sensory neuron-stem cell crosstalk is crucial for maintaining normal physiological functions, with sensory nerves primarily influencing stem cells through neuropeptides (Kline and Yu, 2009). Zhang et al. found that sensory nerves release substantial amounts of calcitonin Gene-Related Peptide (CGRP) near injury sites (Wang et al., 2024). CGRP acts directly on DPSCs via the receptor activity-modifying protein 1 (RAMP1), promoting collective migration of DPSCs to the injury site and ultimately enhancing pulp repair. Additionally, CGRP signaling can control a neuro-vascular-reparative dentin network to promote pulp injury repair (Zhan et al., 2024). However, the sensory neural network, comprising nearly 40% of the pulp volume, likely functions far beyond pain perception (Zhan et al., 2021). It may serve as an “early warning system” for tissue damage and an “active regulator” of the repair process (Byers and Taylor, 1993). By integrating single-cell transcriptomics, *in vitro* functional assays, and animal models, this study systematically reveals the central role of the key neuropeptide CGRP, released from sensory nerve terminals, in dental pulp injury repair. It elucidates the molecular mechanisms by which CGRP drives the repair process through orchestrating a multifaceted “neuro-vascular-stromal cell” communication network. Our study first provides theoretical support for the active regulatory role of sensory nerves through bioinformatics analysis. RNA sequencing of injured pulp tissue showed significant activation of neuroinflammation and vascular development pathways, suggesting functional coupling between the nervous and vascular systems in the injury response. More importantly, re-analysis of public single-cell sequencing data revealed dense ligand-receptor interactions between sensory neurons and endothelial cells, as well as dental pulp cells/fibroblasts within the pulp microenvironment, with the CGRP signaling pathway being particularly prominent. This finding positions sensory nerves as key nodes in the intercellular communication network of the pulp. Notably, in a state of pulpitis, the expression of the CGRP receptor is significantly upregulated, strongly implying that CGRP signaling is specifically activated under pathophysiological conditions. This suggests it is a key component of the damage repair response, rather than a mere concomitant phenomenon.

At the mechanistic level, a key finding of this study is that CGRP promotes the repair process through two synergistic pathways: directly acting on endothelial cells and indirectly by modulating the paracrine function of stromal cells. In the direct pathway, we confirmed that CGRP significantly enhances the tube-forming ability and migration speed of HUVECs. The molecular basis lies in the upregulation of key angiogenic factors such as CD31 and VEGFA. This *in vitro* finding was strongly corroborated in a mouse model of pulp injury. Treatment with the CGRP receptor antagonist BIBN4096BS significantly inhibited angiogenesis in the injury area, confirming the indispensable role of endogenous CGRP signaling in the reparative vascularization of the pulp. Angiogenesis is not only a channel for delivering nutrients and oxygen but also an important platform for recruiting stem cells and transmitting signals (Zhu et al., 2020; Ramasamy et al., 2014). Furthermore, our study found that CGRP-activated endothelial cells can release pro-mineralization factors like BMP2 and TGFβ in a paracrine manner, thereby indirectly enhancing the mineralization capacity of DPSCs (Kong et al., 2024). This finding elegantly explains why some studies observed an inhibitory effect of CGRP on DPSCs in direct assays, while a promotive effect is seen in the overall repair context (Michot et al., 2020). CGRP likely creates a microenvironment conducive to stem cell differentiation by modulating vascular endothelial cells as an “intermediate”. In the indirect pathway, this study thoroughly investigated the regulatory effect of CGRP on DPCs, the core stromal cells of the pulp. We found CGRP to be an effective promoter of DPC migration. RNA sequencing analysis revealed that the transcriptional reprogramming induced by CGRP is highly targeted, being significantly enriched in pathways related to cell migration, movement, and cytoskeletal reorganization. This not only explains the pro-migration phenotype at the genetic level but also suggests that CGRP, by activating signaling pathways like IL-17 and TNF, might be involved in the recruitment of DPCs to the injury site and the formation of repair matrices during the early stages of damage. More critically, CGRP treatment significantly upregulated the expression of VEGFA in DPCs. Functional experiments demonstrated that conditioned medium from CGRP-pretreated DPCs significantly enhanced the tube-forming ability of endothelial cells. This indicates that activated DPCs, by secreting factors like VEGFA in a paracrine manner, feedback into the vascular system, forming a positive feedback loop of “nerves activating stromal cells, and stromal cells supporting angiogenesis”. As the predominant cell population in the pulp tissue, DPCs act as a “signal amplifier” bridging different processes, converting local neural signals into widespread microenvironmental signals, thereby amplifying the repair effect. Our findings, together with the recent report by Wang et al. on CGRP-mediated DPSC migration, paint a more comprehensive picture of sensory nerve orchestration in pulp repair (Wang et al., 2024). Wang et al. elucidated a direct neuro-stem cell axis essential for progenitor recruitment. In parallel, our study reveals a neuro-stromal-vascular axis, wherein CGRP activates pulp fibroblasts to secrete pro-angiogenic factors like VEGFA, thereby fostering a vascular niche conducive to repair. These two axes likely operate synergistically, ensuring both the recruitment of reparative cells (DPSCs) and the timely restoration of blood supply, ultimately leading to effective tissue regeneration.

In conclusion, this study proposes a new paradigm for dental pulp injury repair: sensory nerves act as the “commander”, releasing the “messenger” CGRP. CGRP simultaneously directs the “engineers” (endothelial cells) to construct vasculature and activates the “logistical support” (DPCs) to provide aid and clear the field. Together, they ultimately support the “builders” (DPSCs) in completing hard tissue repair. This “neuro-vascular-stromal cell” multicellular communication network model significantly deepens our understanding of the pulp’s intrinsic repair capacity. This model aligns well with recent advances in pulp regeneration technology. Research has found that the key to successful pulp regeneration lies in creating a suitable microenvironment that promotes the synergy of blood vessels, nerves, and stromal cells. Particularly in the pulp regeneration therapy of young permanent teeth, reconstructing this neuro-vascular network using stem cell technology and tissue engineering approaches has become an important strategy. Our research provides a mechanistic explanation for these clinical observations and emphasizes the therapeutic potential of targeting neural-microenvironment interactions.

## Data Availability

The original contributions presented in the study are included in the article/Supplementary Material, further inquiries can be directed to the corresponding author.

## Supplementary material

SUPPLEMENTARY FIGURE S1 Response to the Reviewer’s Comments. Histological assessment of the pulp-dentin complex response at 7 days after dentin injury. Representative hematoxylin and eosin (H&E)-stained sections of first molars are shown. Mice subjected to dentin injury without further treatment (NC group). Mice subjected to dentin injury followed by systemic administration of the CGRP receptor antagonist BIBN4096 (BIBN group). The depressed area indicate the boundary of the prepared cavity. Scale bar: 50 μm.

SUPPLEMENTARY FIGURE S2 Response to the Reviewer’s Comments. Marker gene expression validates cell type identification in human dental pulp scRNA-seq data. Dot plots (bubble charts) visualize the expression of canonical marker genes across the major cell clusters identified in the integrated and comparative pulp analyses.

SUPPLEMENTARY FIGURE S3 Response to the Reviewer’s Comments. Comparative cellular composition of healthy and inflamed human dental pulp. Bar chart depicting the relative proportions of major cell populations identified by single-cell RNA sequencing. For each cell type indicated on the x-axis, the total height of the stacked bar represents 100% of that cell population across the combined healthy and pulpitis samples. The red segment of each bar indicates the proportion contributed by cells from healthy pulp, while the blue segment indicates the proportion from pulpitis tissue, as defined in the legend.
