# Supplementary figures and images for: CGRP-mediated neuro-vascular-pulp cell crosstalk is essential for dental pulp repair
Source: Front Cell Dev Biol. 2026 Apr 21;14:1793692. doi: 10.3389/fcell.2026.1793692 (PMC13141303; doi:10.3389/fcell.2026.1793692)

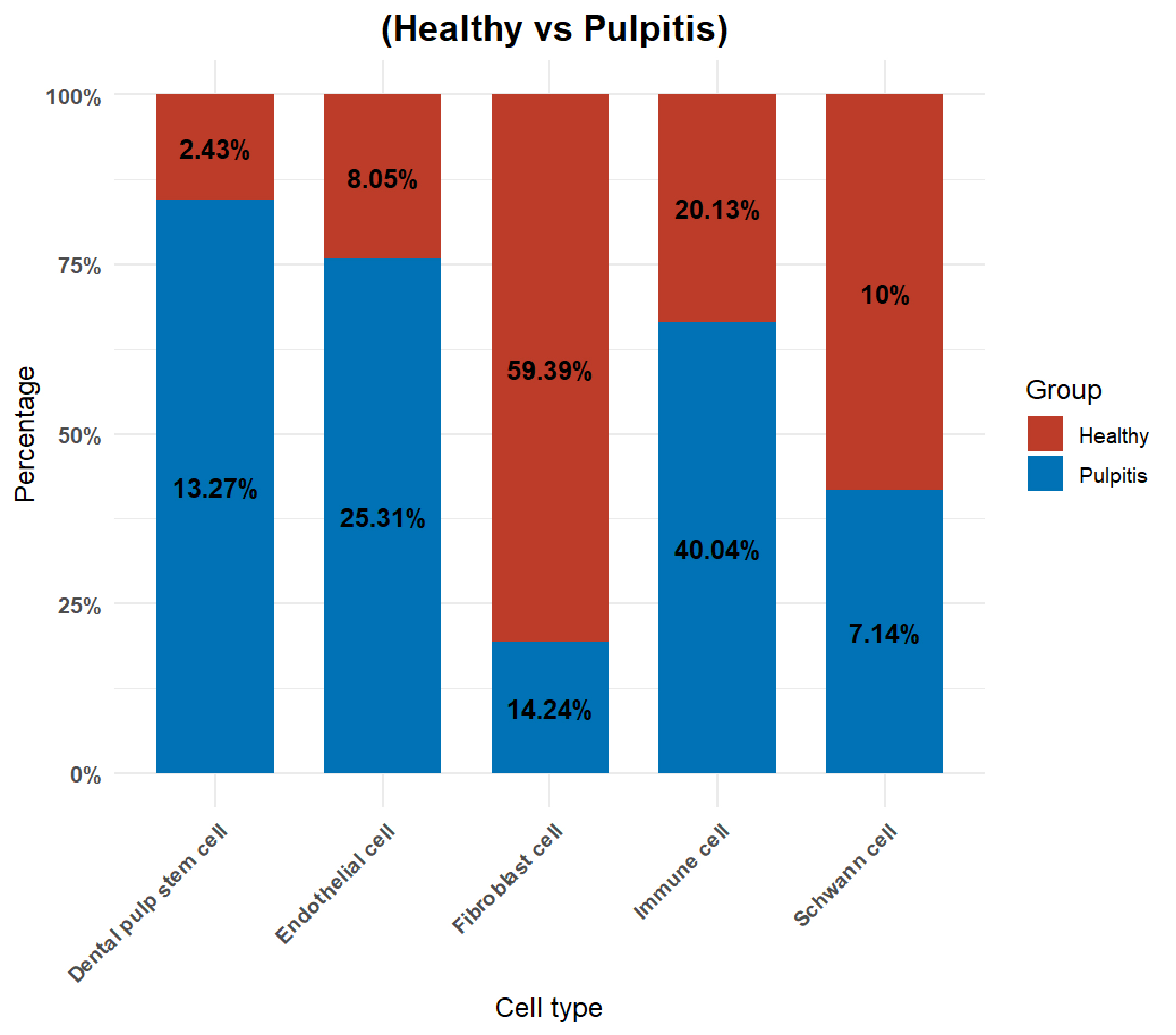

Supplement: Supplementary file 1 [file Image3.jpeg]

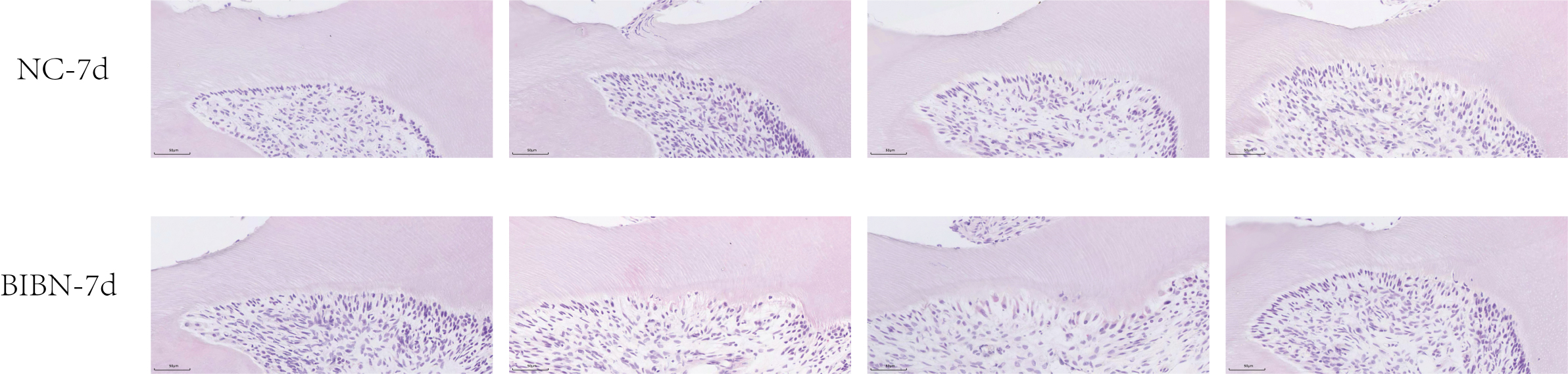

Supplement: Supplementary file 2 [file Image1.jpeg]

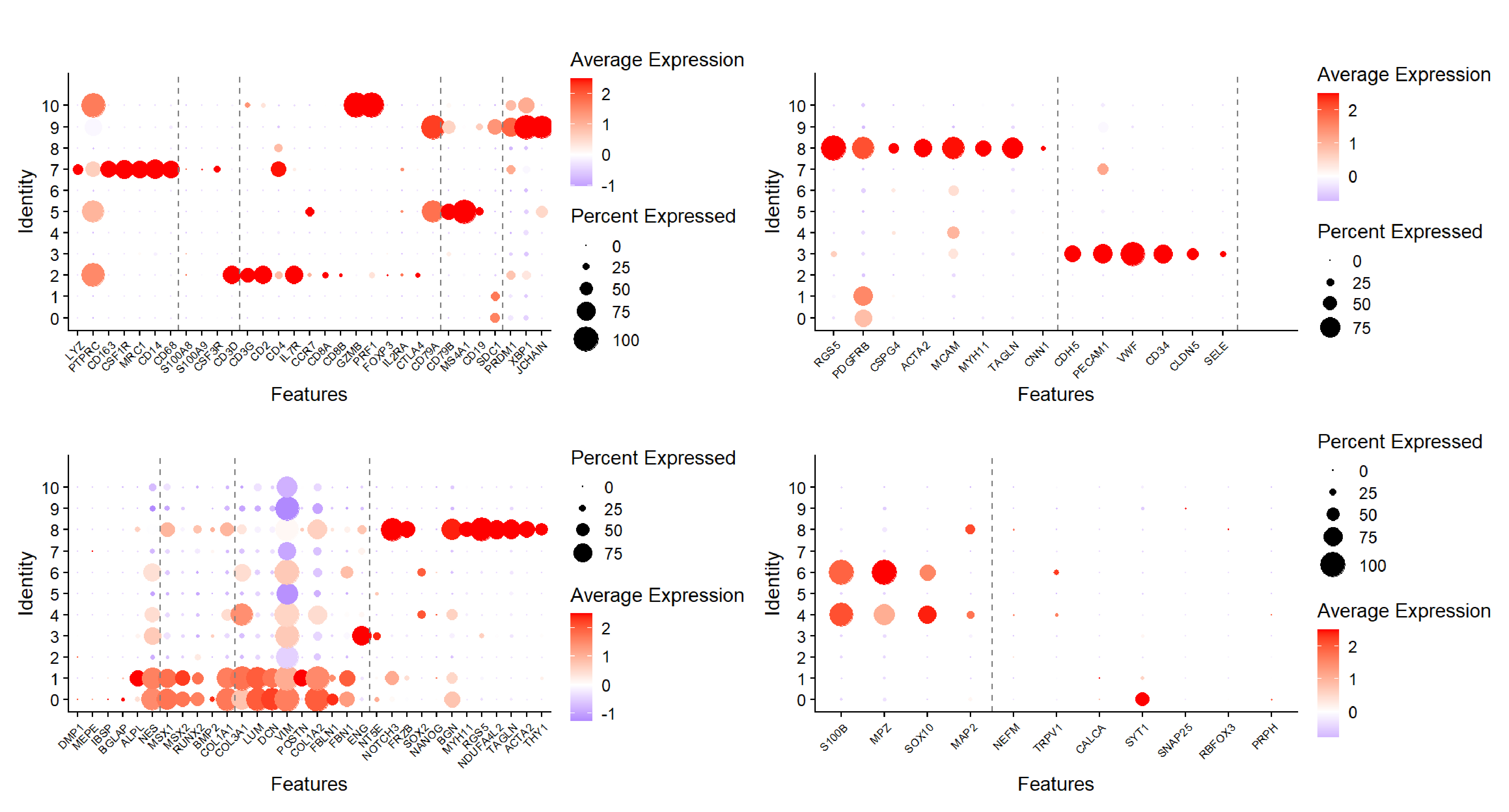

Supplement: Supplementary file 3 [file Image2.jpeg]
